# Fish muscle hydrolysate obtained using largemouth bass *Micropterus salmoides* digestive enzymes improves largemouth bass performance in its larval stages

**DOI:** 10.1371/journal.pone.0261847

**Published:** 2021-12-28

**Authors:** Karolina Kwasek, Christian Gonzalez, Macdonald Wick, Giovanni S. Molinari, Michal Wojno

**Affiliations:** 1 Center for Fisheries, Aquaculture, Aquatic Sciences, School of Biological Sciences, Southern Illinois University, Carbondale, IL, United States of America; 2 Department of Animal Science, The Ohio State University, Columbus, OH, United States of America; National Cheng Kung University, TAIWAN

## Abstract

The present study utilized digestives tracts from adult largemouth bass (LMB) to hydrolyze Bighead carp muscle and obtain an optimal profile of muscle protein hydrolysates that would be easily assimilated within the primitive digestive tract of larval LMB. Specifically, muscle protein source was digested for the larva using the fully developed digestive system of the same species. The objectives of this study were: 1) to develop an optimal *in vitro* methodology for carp muscle hydrolysis using LMB endogenous digestive enzymes, and 2) to evaluate the effect of dietary inclusion of the carp muscle protein hydrolysate on LMB growth, survival, occurrence of skeletal deformities, and whole-body free amino acid composition. The study found that the *in vitro* hydrolysis method using carp intact muscle and LMB digestive tracts incubated at both acid and alkaline pH (to mimic digestive process of LMB) yielded a wide range of low molecular weight fractions (peptides), as opposed to the non-hydrolyzed muscle protein or muscle treated only with acid pH or alkaline pH without enzymes from LMB digestive tracts, which were comprised of large molecular weight fractions (polypeptides above 150 kDa). Overall, the dietary inclusion of the carp muscle hydrolysate improved growth performance of larval LMB in terms of final average weight, weight gain, DGC, SGR, and body length after 21 days of feeding compared to fish that received the diet based on non-hydrolyzed carp muscle. The study also found that hydrolysate-based feed significantly reduced skeletal deformities. The positive growth performance presented by fish in the hydrolysate-fed group possibly resulted from matching the specific requirements of the larvae with respect to their digestive organ development, levels of digestive enzymes present in the gut, and nutritional requirements.

## Introduction

The successful growth and survival of many fish species, particularly at their young stage, strongly depends on environmental conditions, namely food availability and water quality, making fish production in outdoor systems (i.e. ponds) often unpredictable. To intensify fish production, aquaculture has been moving towards sustainable farming intensification, which utilizes indoor recirculation systems. Such technology, however, poses a challenge related to the lack of naturally occurring live food that larval fish generally prey on. Therefore, the substitution of live food with formulated diets for the early stages of fish has become a major focus in aquaculture during the last decades in order to reduce costs and increase predictability of juvenile production. Different types of formulated feeds have been examined from dry or frozen live food organisms, feeds supplemented with digestive enzymes, to formulated food particles made using different processing technology and methods [1–3]. However, weaning first feeding larval fish completely onto formulated dry feeds has not been fully possible yet.

The larval digestive tract is often not completely developed during the first weeks of life, and therefore digestive processes, protein digestion in particular, significantly limit the utilization of formulated feeds by larval fish. The growth of young fish is rapid and consequently requires delivery of amino acids (AA)—protein building blocks, in a highly available form for both energy and tissue protein synthesis [4]. These dietary AA can be provided in different molecular forms: protein-bound, free AA (FAA), or peptides, and all induce different responses in larval fish [5, 6]. For example, free AA-based diets are not well utilized for protein synthesis and growth [7, 8], while peptide-based diets seem to support good growth performance in some species [9, 10]. Various protein hydrolysates have been obtained using *in vitro* methods that have attempted to reproduce the physiological conditions of the digestive tract [11]. However, to date the practical application of *in vitro* hydrolysis has not been routinely used by the feed industry due to complexity and low repeatability.

Live food commonly used in larval fish indoor rearing protocols contains a substantial amount of soluble nitrogen in the form of peptides and free AA [12, 13]. Since live food undergoes autolysis in the fish gut, these low molecular weight fractions increase substantially shortly after ingestion [1]. On the other hand, formulated diets contain higher molecular weight, intact proteins that are difficult for larvae to digest compared to live food. As a result, poor growth is associated with low digestion and assimilation of dry (formulated) feeds. It is widely known that the inclusion of pre-digested protein in the form of protein hydrolysates improves larval growth performance [14–16]. However, larval capacity to digest dietary components of different molecular weights changes throughout its development [17] and therefore, the right balance between different sizes of protein fractions in diets is critical to induce positive growth responses in larval fish.

“Asian carp” mostly refers to Silver Carp (*Hypophthalmichthys molitrix*) and Bighead Carp (*H*. *nobilis*) species—an invasive species, which, in the last few decades, have threatened the Great Lakes via their uncontrollable dispersion from previously established populations. Following this rapid increase of Asian carp and the absence of natural barriers to prevent further spread of this invasive fish species, several management strategies including physical, behavioral, and chemical barriers, have been established aimed at limiting Asian carp movement and reducing their density. Harvest has also been considered as an approach to reducing Asian carp abundance, however, considering that Asian carp are not favored food fish in the US, finding a local market for the fish has been a challenge. There has been increased interest, however, in the last few years, to utilize Asian carp to produce fishmeal, a protein ingredient commonly used in production of aquaculture diets. This would be particularly reasonable, since replacement of fishmeal derived mainly from marine pelagic fishes with alternative protein sources has been considered a major bottleneck to further expansion of the aquaculture industry for decades. Asian carp has been shown to be a suitable replacement for marine fishmeal without compromising growth of largemouth bass (LMB) (*Micropterus salmoides*) [18]. In fact, Asian carp body composition has been reported to be similar to traditional, more expensive marine fishmeal sources [19]. Furthermore, Mallaypally et al. [20] suggested the potential use of Asian carp as a source of protein hydrolysate and antioxidants, providing an alternative application to the use of these invasive species as a functional health-promoting ingredient. This study proposes the next important step in promoting the use of Asian carp as a fishmeal replacement by evaluating Asian carp muscle as an initial protein source for early stages of LMB in the form of high-quality protein hydrolysate. Specifically, we propose to utilize digestives tracts from adult LMB in order to hydrolyze Asian carp muscle and obtain optimal profile of muscle protein hydrolysates that will be easily assimilated within the primitive digestive tract of larval LMB. In other words, we propose to digest the muscle protein source for the larva using fully developed digestive system of the same species.

The objectives of this study were as follows: 1) To develop the optimal *in vitro* methodology for Asian carp muscle hydrolysis using LMB endogenous digestive enzymes obtained from adult LMB; and 2) To evaluate the effect of Asian carp muscle protein hydrolysate obtained using the methodology in Objective 1 as a replacement for non-hydrolyzed protein in larval LMB diets on growth, survival, occurrence of skeletal deformities, and muscle postprandial FAA composition used as an indicator of dietary AA availability.

## Materials and methods

### Ethics statement

The feeding trial was conducted in the Center for Fisheries, Aquaculture, and Aquatic Sciences at Southern Illinois University-Carbondale (SIUC), IL. All experiments were carried out in strict accordance with the recommendations in the Guide for the Care and Use of Laboratory Animals of SIUC. The SIUC Institutional Animal Care and Use approved all of the protocols performed (protocol #18–051). All researchers were trained in accordance with SIUC Institutional Animal Care and Use requirements. During fish handling, euthanasia and anesthesia were performed using water bath immersion in tricaine methanesulfonate (MS222) at a recommended concentration, and all efforts were made to minimize pain, stress, and discomfort in the animals. Humane endpoints were used in this study, fish were euthanized within 24 hours if they showed significant signs of deteriorating quality of life. Fish were observed each day for signs of distress.

### Muscle hydrolysis using digestive enzymes from adult largemouth bass

Bighead carp were captured from the upper part of the Illinois River (Marseilles, IL) and freshly sampled muscles were transported on ice to the SIUC laboratory and stored at -80°C until further processing. Thawed muscles were ground three times with a meat grinder (General Food Service, Weston, FL) and homogenized with a PowerGen 1000 (Fisher Scientific, Waltham, MA) tissue homogenizer on high speed for ten minutes.

Adult LMB were kept at 22°C and fed at 3% feeding rate (based on fish biomass). On the day of the hydrolysis, the fish were fed at approximately 1% feeding rate (one meal) and after approximately 2 hours, the fish were humanely euthanized with an excess of MS222 and their digestive tracts were dissected and placed on ice. The 2-hours after a meal was a time established prior to the experiment which guaranteed digestion of the provided pelleted feed (and hence, release of digestive enzymes) as well as presence of the feed still in the gut (before full fecal excretion of the feed and endogenous enzymes). The digestive tracts were homogenized, and the homogenates were subsequently centrifuged (1500 x g for 10 minutes at 4°C) to obtain the supernatant and to separate it from the solid mass (this included undigested feed, fat, and other tissues). The supernatant was immediately used for further muscle hydrolysis.

**Fig 1:**
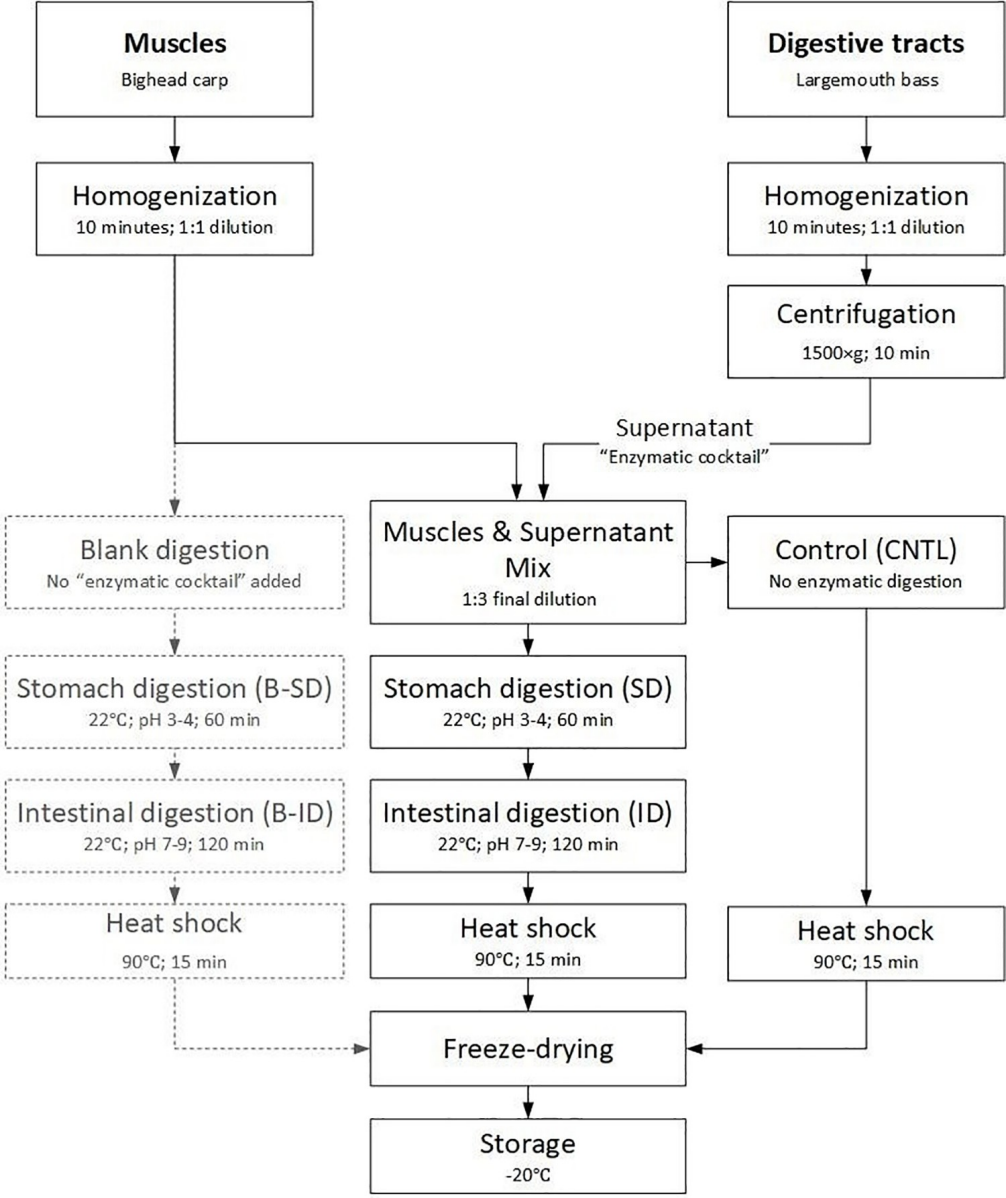
Muscle hydrolysis method. An illustration of methodology for hydrolysis of Bighead carp muscle using endogenous digestive enzymes from adult LMB.

The Bighead carp muscle homogenates were moved to 12-liter containers placed in a water bath, diluted further with deionized water (1:3), stirred using an overhead stirrer (VWR VOS 16), and divided into three portions. One portion of the muscle homogenate was hydrolyzed (muscle hydrolysate), another portion served as a control (non-hydrolyzed muscle), and the remaining served as a blank. For the hydrolysis, after temperature and pH had been adjusted to the required level, carp muscle homogenates were mixed with LMB digestive tract supernatants (22°C; initial pH 3–4 for the first one hour to mimic stomach digestion followed by pH 7–9 for two hours to mimic intestinal digestion [21]). The incubation temperature was set at 22˚C. Stomach pH was adjusted using 3M HCL, while intestine pH was adjusted using 2.5 M NaOH. Each digestive tract supernatant was mixed with carp muscle in a ratio that represented protein content on a dry feed basis that would be consumed by LMB of adult size (~1–1.5 pound) in one day at ~3% feeding rate of commercial feed. This translated into 1:1.5 digestive tract to muscle ratio (on a wet weight basis). For the control, Bighead carp muscle homogenate and digestive tract supernatant mix were both incubated at 90°C for 15 minutes immediately after mixing to inactivate enzymatic activity. For the blank, carp muscle homogenate without the addition of digestive tract supernatant was incubated in parallel with muscle hydrolysates in the exact same conditions (pH, temperature, and time duration). The incubation process was terminated by increasing the temperature to 90°C for 15 min for both muscle hydrolysate and blank to deactivate any digestive tract and/or muscle endogenous enzyme activity. The muscle hydrolysates, non-hydrolyzed muscle (control), and blank were stored at -80°C and later freeze-dried for subsequent analyses and inclusion in the feed (blank was not included in any of the experimental feeds and was solely used to help assess the degree of the hydrolysis). The hydrolysis process is depicted in Fig 1.

### SDS-PAGE

Electrophoretic analysis of the samples was conducted with sodium dodecyl sulfate-polyacrylamide gel electrophoresis (SDS-PAGE) with modifications of the method described by Updike et al. [22]. The SDS-PAGE was performed in order to present the products of muscle hydrolysis. Samples were dissolved in dissociation buffer (8 M urea, 2M thiourea, 60 mM Tris buffer, pH 6.8, containing 2% SDS, 15% glycerol, 350 mM DTT, and 0.1% bromophenol blue) overnight at 25°C with agitation, then diluted with dissociation buffer as needed. Approximately 10 μl of sample dilutions were loaded onto each lane of a 10% T denaturing resolving gel (30:0.8, acrylamide: N, N’- bis-methylene acrylamide) with a 3% stacking gel containing 1% SDS. The proteins were resolved at 100 V cm-1 until the dye front reached the bottom of the gel. Gels were stained with Coomassie Brilliant Blue G-250 overnight and then destained overnight with 10% acetic acid. After staining and destaining, gels were scanned and the images digitized.

### Diet preparation

For the feeding trial, diets were formulated and produced using Bighead carp muscle as a protein source: an intact (non-hydrolyzed muscle) protein-based feed (Control feed) and 50% muscle hydrolysate-based feed (Hydro feed) (Table 1). The key difference between the two feeds was that the control feed did not contain any carp muscle hydrolysate and was based solely on an intact protein source (74% Bighead carp meal). Whereas the Hydro feed was composed of 37% Bighead carp meal and 37% Bighead carp hydrolysate (1:1 ratio [23]). All diets were formulated to meet nutritional requirements of larval fish [21].

**Table 1:** Formulations and proximate and amino acid composition of experimental diets.

		Diet designations	
		Control	Hydro
*Ingredients*			
	Carp intact muscle meal	74.00	37.00
	Carp muscle hydrolysate		37.00
	CPSP 90^a^	5.00	5.00
	Krill meal^b^	5.00	5.00
	Fish oil^c^	4.00	4.00
	Lecithin^d^	4.00	4.00
	Mineral mix^e^	3.00	3.00
	Vitamin mix^f^	3.00	3.00
	CaHPO_**4**_	1.00	1.00
	Taurine	1.00	1.00
	Choline chloride	0.10	0.10
	Vitamin C^g^	0.05	0.05
	Sum	100	100
*Analyzed composition (g/100 g*, *dry matter)*			
	Crude protein (N x 6.25)	62.10 ± 0.18	61.95 ± 0.62
	Crude lipids	16.48 ± 0.46	16.17 ± 0.78
	Ash	10.44 ± 0.21	10.49 ± 0.11
*Amino acids concentration (g/100g*, *dry matter)*			
	Asparagine/aspartic Acid	5.89 ± 0.09	5.73 ± 0.07
	Glutamine/glutamic Acid	8.58 ± 0.19	8.18 ± 0.11
	Threonine	2.62 ± 0.05	2.57 ± 0.03
	Serine	2.19 ± 0.05	2.14 ± 0.02
	Proline	2.45 ± 0.14	2.49 ± 0.04
	Glycine	3.35 ± 0.05	3.26 ± 0.04
	Alanine	3.54 ± 0.06	3.44 ± 0.05
	Cysteine	0.62 ± 0.01	0.59 ± 0.01
	Valine	3.16 ± 0.07	3.11 ± 0.05
	Methionine	1.70 ± 0.02	1.59 ± 0.02
	Isoleucine	2.96 ± 0.06	2.88 ± 0.05
	Leucine	4.78 ± 0.08	4.63 ± 0.06
	Tyrosine	2.09 ± 0.02	2.03 ± 0.01
	Phenylalanine	2.57 ± 0.03	2.50 ± 0.03
	Lysine	5.61 ± 0.08	5.37 ± 0.08
	Histidine	1.72 ± 0.02	1.62 ± 0.02
	Arginine	3.68 ± 0.06	3.32 ± 0.04
	Tryptophan	0.69 ± 0.01	0.67 ± 0.01
	Taurine	1.40 ± 0.05	1.18 ± 0.02
	Sum	59.60 ± 1.05	57.31 ± 0.68

^a^ Soluble fish protein hydrolysate (Sopropeche S.A., Boulogne Sur Mer, France).

^b^ Processed *Euphausia superba* (Florida Aqua Farms, Dade City, FL, USA).

^c^ Cod liver oil (MP Biomedicals, Solon, OH, USA).

^d^ Refined soy lecithin (MP Biomedicals, Solon, OH, USA).

^e^ Bernhart-Tomarelli mineral mix with 5ppm selenium in a form of sodium selenite (Dyets, Bethlehem, PA, USA).

^f^ Custom Vitamin Mixture (mg/kg diet) Thiamin HCl, 6.84; Riboflavin, 7.2; Pyridoxine HCl, 10.29; Niacin, 16.35; D-Calcium Pantothenate, 75.84; Folic Acid, 1.89; D-Biotin, 0.24; Vitamin B12 (0.1%), 30; Vitamin A Palmitate (500,000 IU/g), 14.49; Vitamin D3 (400,000 IU/g), 12.39; Vitamin E Acetate (500 IU/g), 198; Menadione Sodium Bisulfite, 3.54; Inositol, 750 (Dyets, Bethlehem, PA, USA).

^g^ L-Ascorbyl-2-Polyphosphate (Argent Aquaculture, Redmond, WA, USA).

For diet production each dry ingredient was first ground to 500 μm with a centrifugal mill (Retsch Haan, Germany). Subsequently, all were then manually sieved through a 250 μm sieve to ensure all particles were of the appropriate and uniform size. All the dry ingredients (excluding lecithin and choline chloride) were added together and mixed for 15 minutes, and the fish oil was then added with the soy lecithin dissolved in the oil. The oil and dry ingredients were mixed again for 15 minutes. Finally, water (~10–15% of total mass of feed) was added with dissolved choline chloride and mixed for another 15 min. Feeds were then slowly processed using an extruder (Caleva Extruder 20, Sturminster Newton Dorset, England) to obtain a proper extrudate size and firmness. Extrudates were then processed using a spheronizer (Caleva, Sturminster Newton Dorset, England) at 600 rpm for 3 min, 1800 rpm for 30 seconds, and then 600 rpm for 2–5 minutes to finish the process. Finally, the spheres were dried using a freeze dryer (Labconco, Kansas City, MO). All dried pellets were sieved to appropriate sizes using a vibratory sieve shaker (Retsch Hann, Germany). All finished feeds were stored in sealed bags at -20°C.

### Fish and rearing system

Larval LMB were obtained from La Salle Hatchery, Illinois, at 4 days post-hatch (dph). In order to acclimate the fish to the experimental semi-recirculated system’s water conditions, bags with the larvae were placed in the system water upon arrival to allow the water in the bag to adjust to the temperature gradually. To accelerate the process, small volumes of water (~250 ml) from the system were poured into the bags every 5–10 minutes. Once the temperature and pH were adjusted, larval fish were distributed into nine half-filled 100L light blue fiberglass tanks at a density of 270 larvae per tank. Three replicate tanks were assigned for each experimental group.

Three dietary regimes were tested in the study, two of which were “Control”—where fish received Control feed and “Hydro”—where fish received the Hydro feed. Both of these groups received experimental diets in a combination with *Artemia* nauplii during the first 10 days of feeding to support survival of the early larval LMB stages. The third regime received Hydro feed only without live food (*Artemia* nauplii) supplementation (“Hydro Only”). The feeding of larval LMB started at 5 dph and all larval fish in each group were fed *ad libitum* minimum five times a day throughout the study. The live food and dry feed were not provided at exactly the same time. At each meal, the dry diet feeding was always provided first followed by Artemia nauplii feeding to ensure high intake of the experimental diets. The feed intake was monitored during the feeding and later by assessing fish gut contents. The experiment was carried out using a semi-recirculated aquaculture system with two mechanical (sand) filters (Pentair, Minneapolis, MN) and two tower trickling bio-filters. Water parameters such as temperature and pH were measured daily in the morning and evening, with an overall average of 22.3±1.11°C and 7.75±0.29 pH throughout the experiment. The salinity was kept between 1 and 3 ppt during the live feeding stage to prolong the viability of the live food [24]. The photoperiod consisted of 10 hours of light and 14 hours of darkness, with the overhead lights on 8:00–18:00. After 10 days, larval fish in the Control and Hydro groups were completely weaned off the live food. The remaining 11 days of feeding were based on dry feed use only.

### Sampling

At the end of the study (21 days), the following production parameters were assessed: final average weight, final average length, weight gain, daily growth coefficient (DGC), specific growth rate (SGR), occurrence of skeletal deformities, and survival. The average weight was calculated for each tank by dividing the final biomass by the number of fish in the tank. Additional growth parameters in this study were calculated using the following formulas:

WeightGain(g)=Finalweight(g)−Initialweight(g)

$$Weight\;Gain\;(\%)=\frac{Weight\;gain\;(g)}{Initial\;weight\;(g)}\times 100$$

$$Daily\;Growth\;Coefficient\;(DGC)=\frac{{Final\;weight}^{⅓}-{Initial\;weight}^{⅓}}{Days}\times 100$$

$$Specific\;Growth\;Rate\;(SGR)=\frac{\ln\left(Initial\;weight\right)-\ln\left(Final\;weight\right)}{Days}\times 100$$

A total of 30 fish from each tank were used to assess the body length. Skeletal deformities were recorded for each individual fish from each tank and included symptoms of common deformities such as: scoliosis, lordosis, head/jaw/operculum and tail deformities. The procedure for skeletal deformity assessment was based on Boglione et al. [25]. The extensive review can be useful if additional information on skeletal deformity typology is needed. Finally, for muscle FAA analysis, five fish from each tank were sampled 3 hours after feeding (to assess FAA postprandial levels [6]) and 24 hours after feeding (to obtain FAA physiological baseline).

### Diet analysis

Proximate composition of diets included quantification of the following: crude protein, crude lipid, moisture, and ash (Table 1). Briefly, samples were analyzed for ash by combustion (550°C for 5 h) in a muffle furnace (Lindberg Blue M, MA), crude protein (N×6.25) using a Leco nitrogen analyzer (Model FP-628, Leco Corporation, St. Joseph, MO), and crude lipid was extracted with chloroform–methanol (2:1, v/v) as described by Folch et al. [26]. The AA profile of each feed was analyzed utilizing the Association of Official Analytical Chemists, International (AOAC) Official Method 999.13 [27] (Table 1). All dietary samples were analyzed in triplicates.

### Free amino acid analysis

Muscle samples of three fish from each tank were combined and homogenized together with 0.1 mol/L HCl in 1:9 (w/v) and spun at 12000×g (4°C, 15 min). Supernatants were collected, filtered (Milipore, 10 kDa cutoff at 15000×g, 4°C, 30 min), and later diluted with 0.1 mol/L HCl (1:19 v/v) containing norvaline and sarcosine (40 μmol/L) as internal standards. Blanks (0.1 mol/L HCl + 40 μmol/L norvaline and sarcosine) and external standards (Sigma acid/neutral and basic AA) were prepared along with the sample preparation. The same concentration of glutamine in 0.1 mol/L HCl as an external standard was prepared and added to the basic AA standard. Free amino acids were quantified using Shimadzu Prominence Nexera—i LC-2040C Plus (Shimadu, Japan) according to the Shimadzu protocol No. L529 with modifications. Free amino acid concentrations (expressed as μmol/kg wet body weight) were calculated in LabSolutions software version 5.92 (Shimadzu, Japan) using internal and external standards.

### Statistical analysis

Results are presented as means (± standard deviation). Since one of the groups was later eliminated from the study, the final data from the two remaining groups was analyzed using a paired t-test. Differences between groups were considered significant at P values < 0.05. Statistical analysis was run using R version 3.5.2 (Boston, MA). The Shapiro-Wilks normality tests was used to determine if each data set presented a normal distribution prior to statistical analyses. The collected data was analyzed under the independent observation assumption.

## Results

### Bighead carp muscle hydrolysates

**Fig 2:**
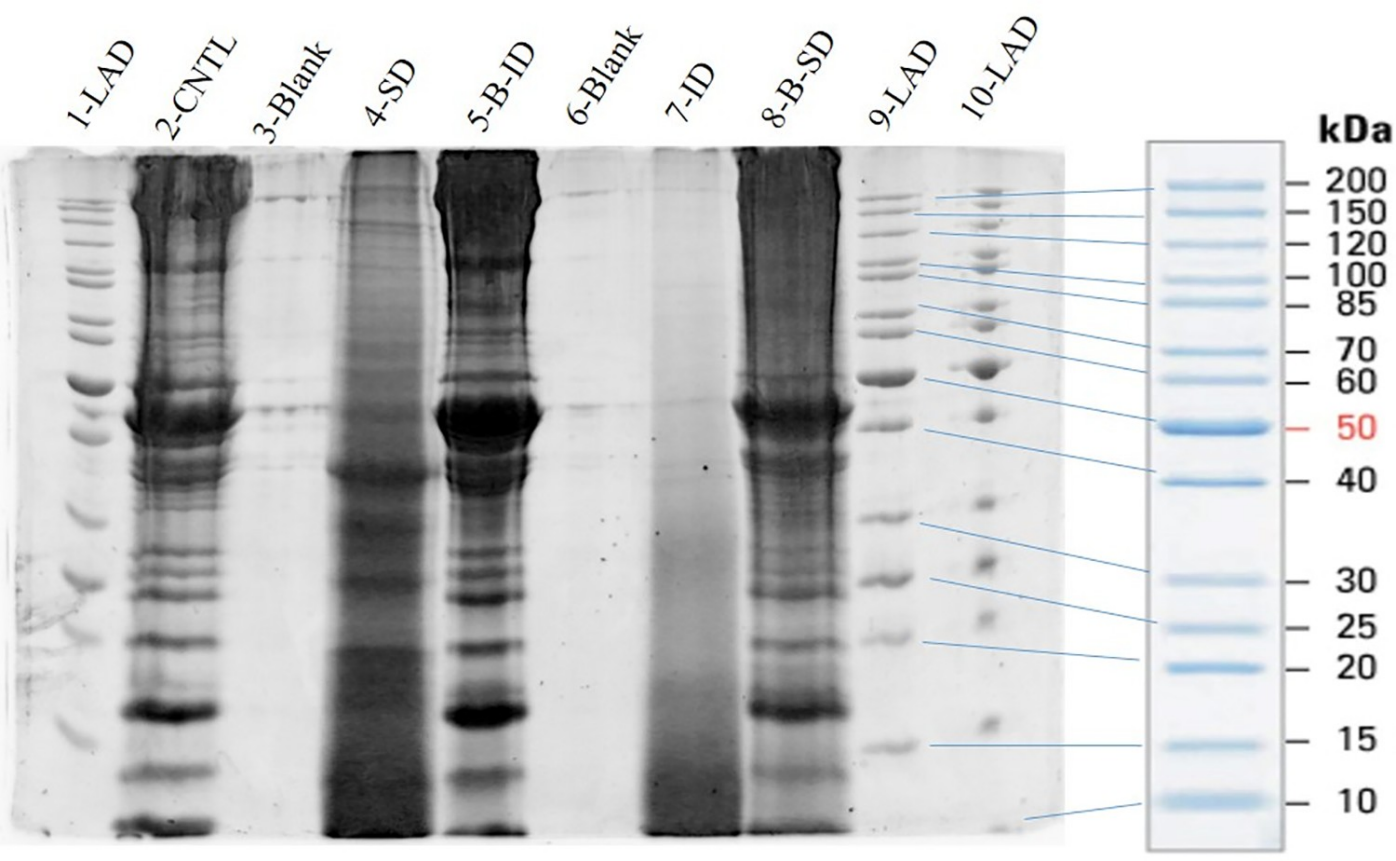
SDS-PAGE of Bighead carp muscle hydrolysate. 10% T denaturing SDS PAGE of Bighead carp muscle hydrolysate obtained using adult largemouth bass endogenous digestive enzymes. The treatments presented are as follows: Lane 1—LAD–protein ladder (marker; 200–10 kDa from top to bottom); Lane 2-CNTL–control, muscle homogenate and enzymatic cocktail mix after heat shock, no enzymatic digestion; Lane 3-Blank–electrophoretic blank sample; Lane 4-SD–stomach digestion, muscles enzymatically hydrolyzed in acid pH; Lane 5-B-ID–blank intestinal digestion, muscles incubated in acid and alkaline pH without LMB digestive enzyme extract; Lane 6-Blank–electrophoretic blank sample; Lane 7-ID–intestinal digestion, muscles enzymatically hydrolyzed in acid and alkaline pH; Lane 8-B-SD–muscles incubated in acid pH without LMB digestive enzyme extract; Lane 9, 10—LAD–protein ladder.

Fig 2 presents the products of Bighead carp muscle hydrolysis obtained using endogenous digestive enzymes from LMB digestive tracts on 10% SDS-PAGE gel. All lanes were loaded with 200 ug of protein based on dry weight of lyophilized protein powder. The results indicate that muscle samples treated with digestive enzymes and incubated in both acid and alkaline conditions (to mimic the digestive process of LMB; Intestinal Digest (ID)) were composed of a range of peptides predominantly migrating at molecular weights lower than 30 kDa, as opposed to the non-hydrolyzed muscle protein (**Lane 2**), or muscle treated only in acid (**Lane 4**) or alkaline (**Lane 8**) conditions without enzymes from LMB digestive tracts. Although no densitometry was performed, the protein/peptide staining in **Lane 7** below ~30 kDa suggests the higher molecular weight proteins observed in **Lane 2** were hydrolyzed into smaller oligopeptides. It is reasonable to expect that the peptides migrating less than 30 kDa, could likely be peptides of less than 275 amino acids (assuming 110 Da/amino acid) with the even lower molecular weight species consisting of single, di- or possibly tri-peptide size. The ID hydrolysate (**Lane 7**) was used in the feeding trial in the Hydro feed. Control (**Lane 2**) was used as the protein source in the Control diet.

### Growth performance

Throughout the feeding trial, both of the experimental diets, Control feed and Hydro feed, were actively ingested and consumed by larval LMB. At the end of the feeding trial, high survival of larval LMB was exhibited in both the Control and Hydro groups and ranged from 55 to 69%, respectively (Table 2). No statistical differences in survival were detected (*p*>0.05). However, larval LMB in the Hydro Only group (fed dry feed only, not supplemented with live food) presented over 90% mortality after 10 days from the trial start and hence, this group was eliminated from the study at that stage.

**Table 2:** Treatment effect on growth performance and survival measures.

	Dietary Treatment	
	Control	Hydro
Survival (%)	55.33 (± 2.07)	69.35 (± 7.33)
Body Length (mm)	20.81^a^ (± 0.50)	23.40^b^ (± 0.57)
Final Average Weight (g)	0.15^a^ (± 0.02)	0.20^b^ (±0.01)
Weight Gain (%)	4873^a^ (± 647)	6341^b^ (± 253)
DGC* (%)	1.56^a^ (± 0.10)	1.76^b^ (±0.03)
SGR** (%)	15.60^a^ (±0.54)	16.66^b^ (±0.16)

Values are presented as means (± std. dev). Superscript letters indicate statistical significance between groups at *p* value <0.05.

*Daily growth coefficient

**Specific Growth Rate.

At the end of the feeding trial, LMB in the Hydro group presented significantly larger final weight and body length compared to the Control group (*p*<0.05; Table 2). In addition, the Hydro group was characterized by significantly higher weight gain compared to the Control group (*p*<0.05; Table 2). Similarly, both DGC and SGR were significantly higher in the Hydro group compared to the Control (*p*<0.05; Table 2).

**Fig 3:**
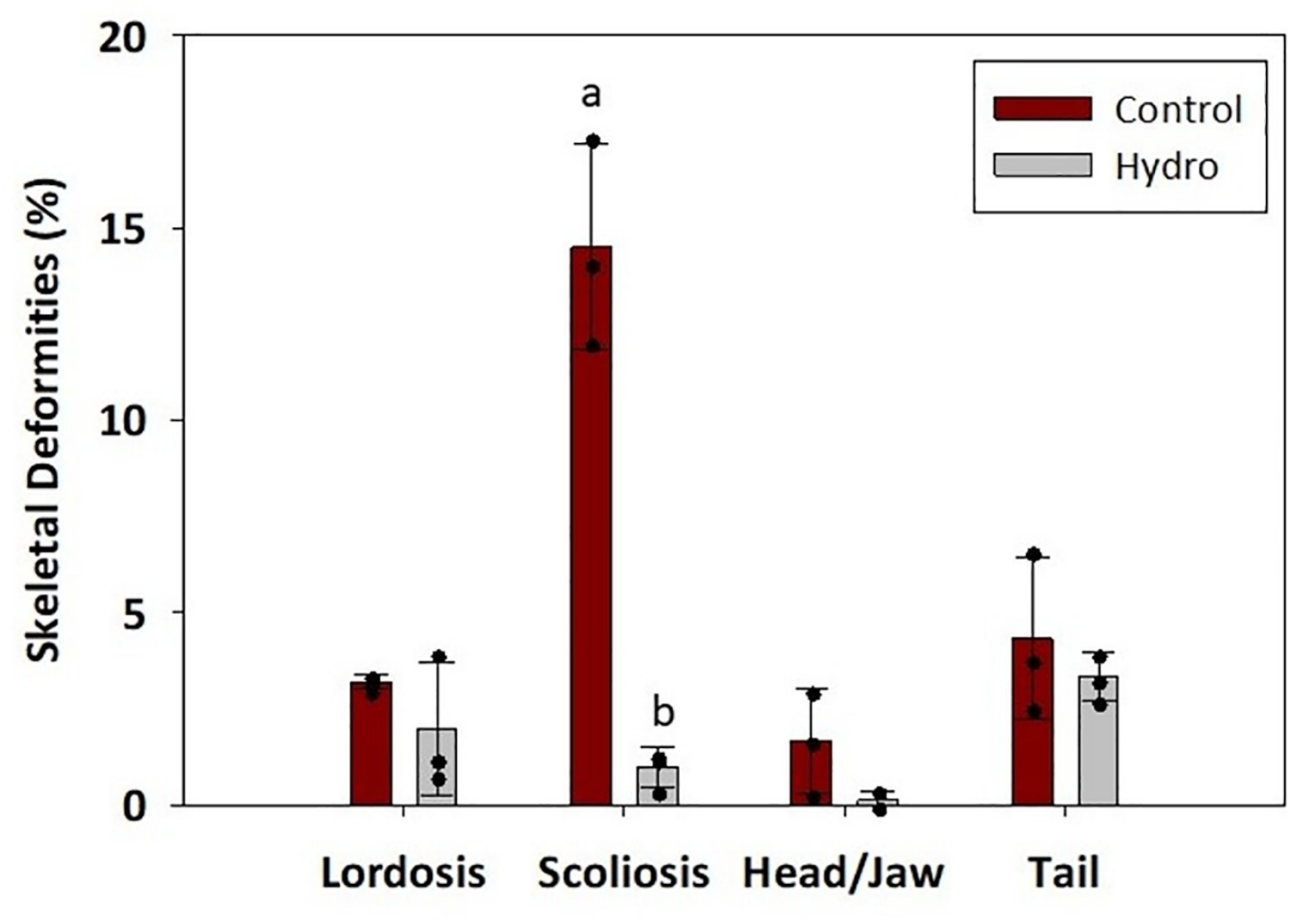
Occurrence of individual skeletal deformities. The individual skeletal deformities in LMB fed control diet (intact muscle protein-based) and Bighead carp muscle hydrolysate-based diet. The black dots represent individual data points from the replicate tanks. Different letters indicate statistical difference at *p*>0.05.

**Fig 4:**
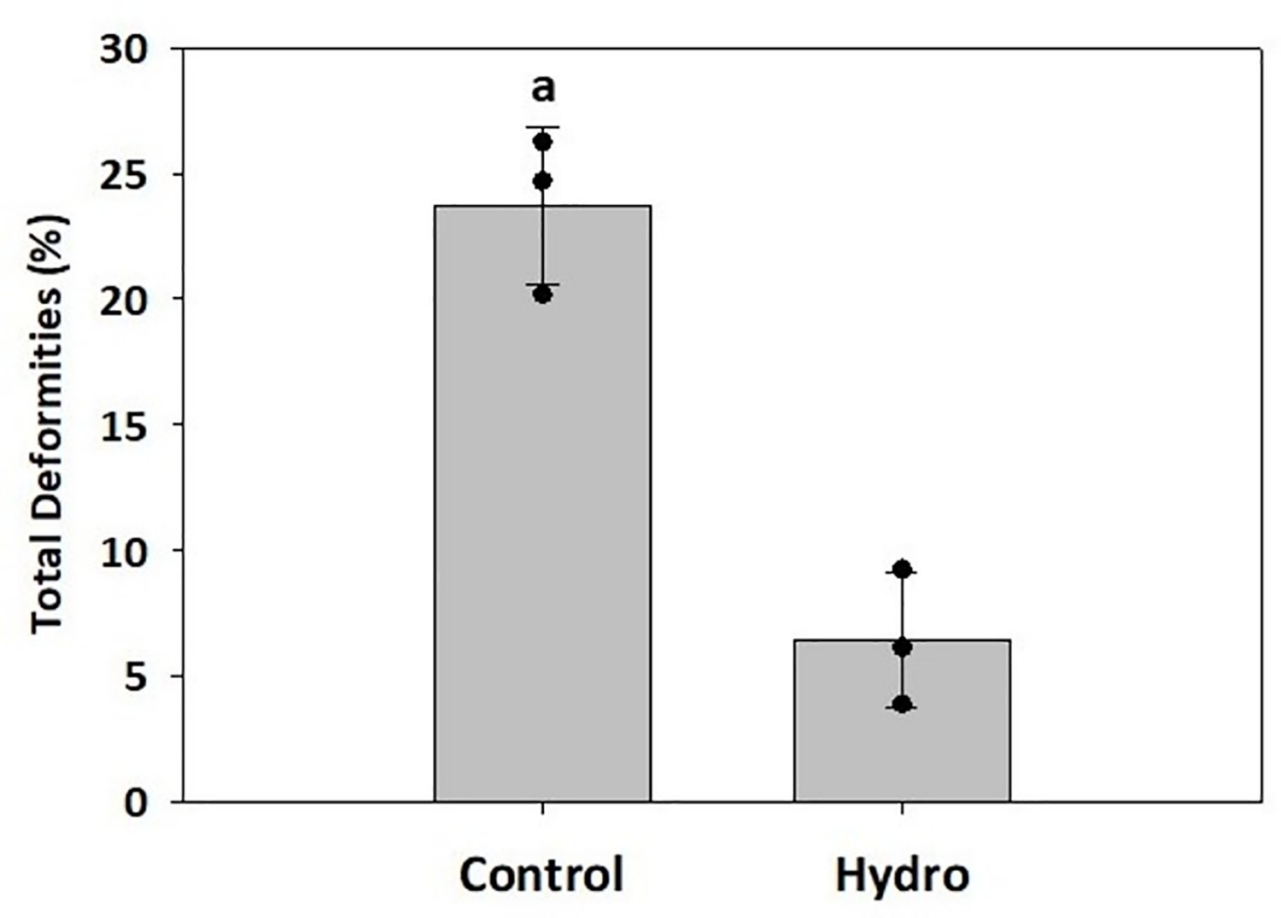
Total skeletal deformities. Percentage of total skeletal deformities in LMB fed control diet (intact muscle protein-based) and Bighead carp muscle hydrolysate-based diet. The black dots represent total skeletal deformities from the replicate tanks. Different letters indicate statistical difference at *p*<0.05.

Finally, no significant differences were detected for the following individual deformities: lordosis, head/jaw, and tail deformities (*p*>0.05; Fig 3). However, the occurrence of scoliosis was significantly higher in the Control versus Hydro group (*p*<0.05; Fig 3). In addition, the total skeletal deformities decreased significantly in the Hydro group compared to the Control (*p*<0.05; Fig 4).

### Muscle free amino acid levels

**Fig 5:**
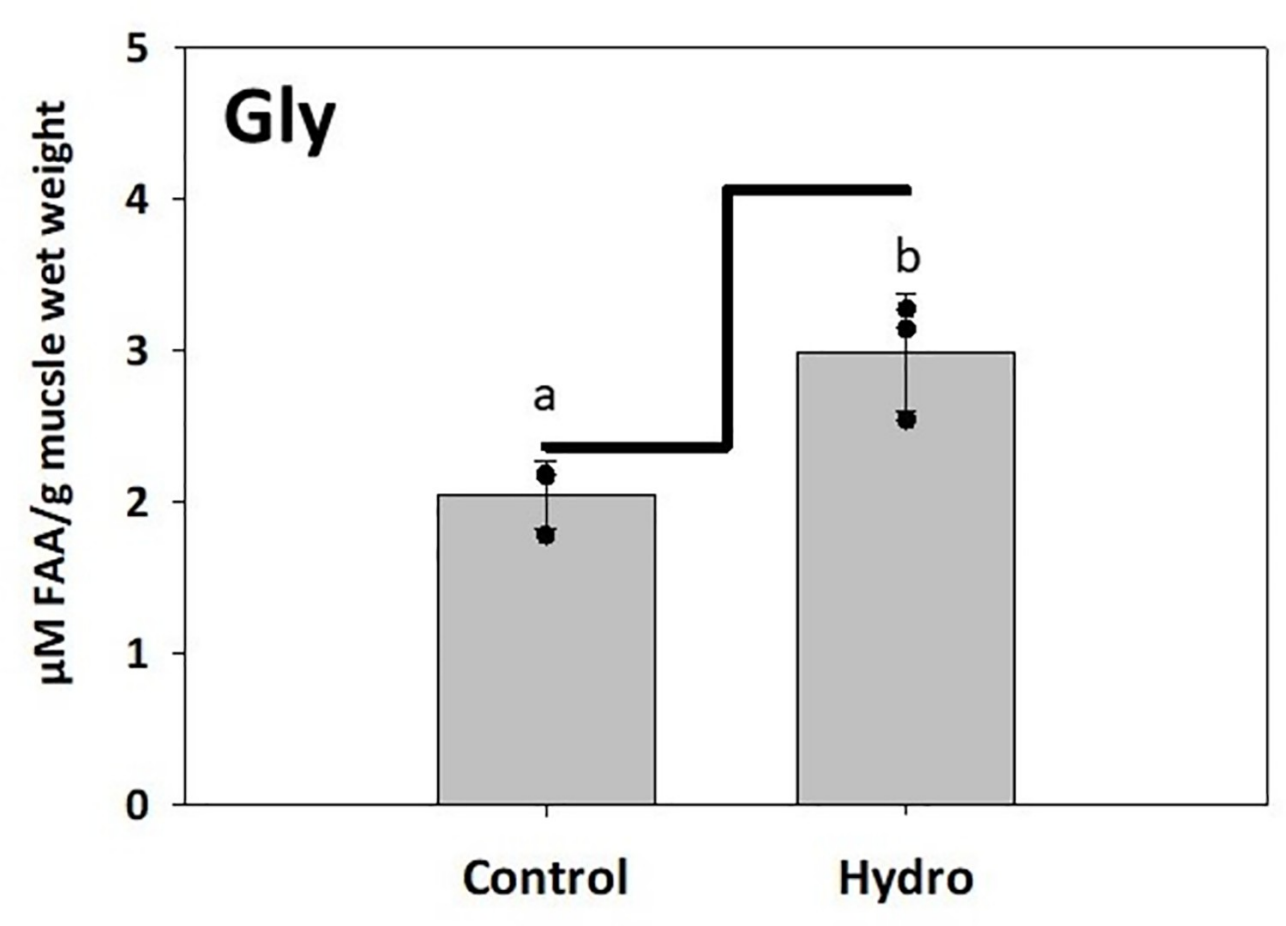
Free glycine levels in LMB muscle. Muscle free glycine levels in LMB after 3 weeks of feeding Control diet (non-hydrolyzed Asian carp muscle-based) and Hydro diet (50% Asian carp muscle hydrolysate-based). The bars represent free glycine level 3-hours after feeding. The line indicates free glycine physiological baseline. The black dots represent average free glycine levels from the replicate tanks. Different letters indicate statistical difference at *p*<0.05.

**Fig 6:**
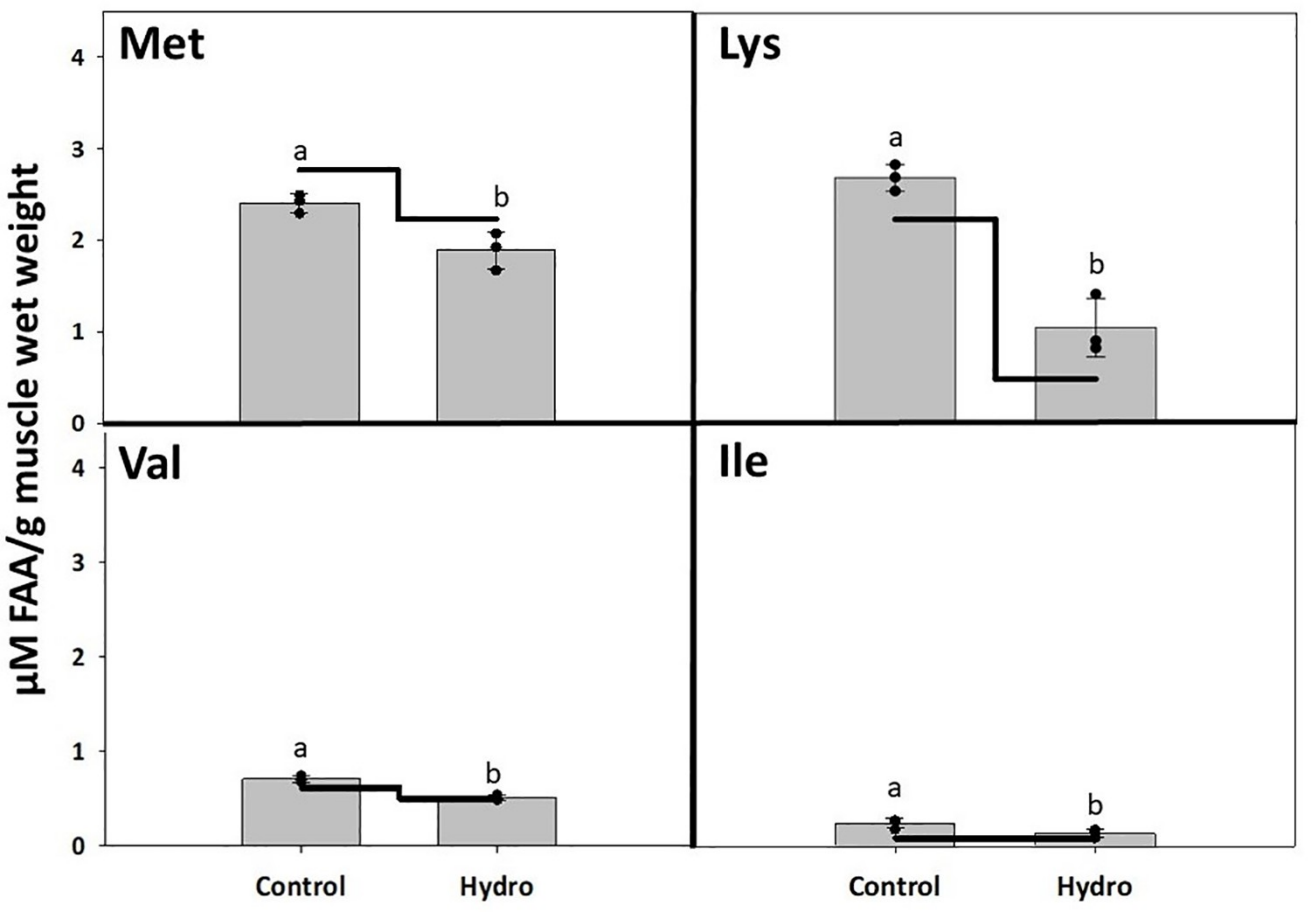
Muscle free amino acid levels in LMB. Muscle free amino acid levels in LMB after 3 weeks of feeding Control diet (non-hydrolyzed Asian carp muscle-based) and Hydro diet (50% Asian carp muscle hydrolysate-based). The bars represent free amino acid levels 3-hours after feeding. The line indicates FAA physiological baseline. The black dots represent the free FAA levels from the replicate tanks. Different letters indicate statistical difference at *p*<0.05.

No significant differences were detected in the level of free indispensable AA (IDAA), dispensable AA (DAA), or total FAA in LMB muscle 3 hours after feeding between the Hydro and Control groups (*p*>0.05; Table 3). The only significant results were detected for free glycine, isoleucine, lysine, methionine, and valine (*p*<0.05). The level of free glycine 3 hours after feeding was significantly lower in the Control versus Hydro group (Fig 5). However, the concentrations of free isoleucine, lysine, methionine, and valine showed an opposite trend with levels significantly lower in the Hydro compared to the Control group (Fig 6).

**Table 3:** Postprandial muscle free amino acid levels in LMB.

Amino Acid (μM/g)	Control	Hydro
Aspartic Acid	0.29 (± 0.04)	0.38 (± 0.07)
Glutamic Acid	1.46 (± 0.10)	1.42 (± 0.12)
Asparagine	0.34 (± 0.06)	0.42 (± 0.10)
Glutamine	1.11 (± 0.21)	0.90 (± 0.28)
Arginine	0.12 (± 0.02)	0.10 (± 0.02)
Histidine	0.68 (± 0.10)	0.55 (± 0.06)
Serine	0.84 (± 0.24)	0.81 (± 0.22)
Threonine	0.99 (± 0.03)	1.02 (± 0.14)
Alanine	1.11 (± 0.08)	1.21 (± 0.08)
Tyrosine	0.14 (± 0.02)	0.11 (± 0.00)
Tryptophan	0.03 (± 0.00)	0.03 (± 0.00)
Phenylalanine	0.12 (± 0.02)	0.11 (± 0.01)
Leucine	0.54 (± 0.12)	0.31 (± 0.10)
Proline	1.19 (± 0.11)	1.14 (± 0.18)

Postprandial muscle free amino acid levels in LMB after 3 weeks of feeding Control diet (non-hydrolyzed Asian carp muscle-based) and Hydro diet (50% Asian carp muscle hydrolysate-based).

## Discussion

Several studies have attempted to quantify the capacity of fish larvae to utilize dietary protein, and the available research suggests that the uptake of protein into the larval body is limited by proteolytic rather than the absorptive capacity of the intestinal tract [28–30]. These reports also agree with previous studies that had indicated a rapid and efficient absorption of low molecular weight protein fraction such as FAA, peptides, and hydrolyzed proteins in larval fish as opposed to intact protein [31–36]. It is generally accepted that larval ability to digest dietary intact protein increases gradually throughout ontogeny in parallel with the ongoing maturation of the digestive tract and subsequent increase in enzymatic activities [17, 28, 36, 37]. More specifically, after metamorphosis is completed, fish go through a process referred to as “enzymatic maturation” of the intestine, characterized by a decrease in cytosolic activity (measured as leucine-alanine peptidase activity) and an increase in the activity of brush border membrane enzymes (measured as aminopeptidase N and alkaline phosphatase activity) [37]. This has not been confirmed in some agastric fish species though [38]. Considering this developmental dynamic of the larval digestive capacity, it becomes clear that protein hydrolysate inclusion in diets can be beneficial. Due to especially high peptidase activity which declines during development, larval fish have the ability to use dietary protein hydrolysates in those early stages better than intact protein. This has been reported in a number of species including goldfish (*Carassius carassius*) [39], carp (*Cyprinus carpio*) [40], sea bass (*Dicentrarchus labrax*) [16, 41], gilthead seabream (*Sparus aurata*) [42], Asian seabass (*Lates calcarifer*) [43, 44], Japanese eel (*Anguilla japonica*) [45], yellow croaker (*Larimichthys crocea*) [46], and Atlantic salmon (*Salmo salar*) [47].

The present study was the first attempt to “digest” protein source for the fish larva using the fully developed digestive system of the same fish species in order to increase dietary AA uptake within the primitive larval digestive tract. Although the present study found that the carp muscle hydrolysate obtained using digestive enzymes from adult LMB did not support the growth and survival of larval LMB during the first feeding when live food was completely eliminated, overall the inclusion of the hydrolysate improved growth performance of larval LMB in terms of final average growth, weight gain, DGC, SGR, and body length after 21 days of feeding as opposed to fish that received a diet based on non-hydrolyzed carp muscle. The positive growth performance presented by fish in the Hydro group possibly resulted from matching the specific requirements of the larvae with respect to their digestive organ development, levels of digestive enzymes present in the gut, and nutritional requirements. Ovissipour et al. [48] and Cahu et al. [15] reported that an incorporation of a moderate dietary level of fish protein hydrolysate promotes the onset of the “adult mode of digestion” in developing Persian sturgeon (*Acipenser persicus*) and sea bass larvae, respectively. However, larval capacity to digest dietary components of different molecular weights changes throughout its development [17]. Our study found that the *in vitro* hydrolysis method using carp intact muscle and LMB digestive tracts incubated at both acid and alkaline pH (to mimic digestive process of LMB) yielded a wide range of low molecular weight fractions (peptides) as opposed to the non-hydrolyzed muscle protein or muscle treated only with acid pH or alkaline pH without enzymes from LMB digestive tracts, which were comprised of large molecular weight fractions (polypeptides above 150 kDa). Furthermore, our data presented on Fig 2 also showed that muscle incubated at stomach pH only with digestive enzymes generated larger molecular weight fragments (SD; lower degree of hydrolysis) as opposed to muscle treated with digestive enzymes and incubated at both acid and alkaline pH (ID; higher degree of hydrolysis) indicating that intestinal enzymes were likely still viable and active during the intestinal digestion. In addition, muscle samples treated with digestive enzymes and incubated at both acid and alkaline pH (ID) were composed of a wide range of lower molecular weight fractions (higher degree of hydrolysis) as opposed to muscle treated only with acid and alkaline pH without enzymes from LMB digestive tracts (B-ID), which suggested that changes in pH alone were not sufficient to induce a high degree of hydrolysis. These results confirmed the efficacy of the method to generate a high-quality protein hydrolysate using whole digestive tracts. However, inclusion of the hydrolysate in the diet was not able to replace the live food use in larval LMB rearing. Since the development of the larval digestive tract is dynamic, it is possible that dietary formulations should change as the larva grows to closely match the continuously maturing digestive tract rather than using a “fixed” dietary formulation from the first feeding until the end of larval metamorphosis (juvenile stage). Canada et al. [17] showed, for example, that Senegalese sole (*Solea senegalensis*) pre-metamorphic larvae are much better at digesting 5–70 kDa oligopeptides compared to metamorphosing and post-larvae that are more efficient in utilizing polypeptides and intact proteins, respectively. In fact, it has been shown that highly hydrolyzed (< 1.4 kDa) and partially-hydrolyzed (10–75 kDa) proteins are absorbed 3.0 and 2.2 times (respectively) faster than intact protein (> 65 kDa) within the first 2 hours after tubefeeding pre-metamorphic Atlantic halibut (*Hippoglossus hippoglossus*) larvae [28], suggesting that the molecular size of the protein fraction is critical to support proper development of larval fish. Studies also indicate that dietary excess of protein hydrolysates can reduce growth performance in some species [15, 42]. For example, Atlantic cod (*Gadus morhua*) larvae performed better with up to 40% hydrolyzed protein in the diet, while Atlantic halibut larvae presented reduced survival with more than 10% hydrolyzed protein [49]. The authors argued that those discrepancies might have arrived from differences in feed consumption rates between species and hence higher or lower potential for leakage of water-soluble hydrolyzed protein from the diet before ingestion. Functional properties of dietary hydrolysates, and therefore, differences in responses in larval fish subjected to protein hydrolysate-based diets, can also vary depending on the peptide composition, protein source and digestive enzymes used for the hydrolysis process, duration of hydrolysis and its conditions [50], as well as level of dietary inclusion [15].

Skeletal deformities in farmed fish occur frequently in rearing conditions, and they represent a major challenge in the aquaculture industry due to not only biological and animal welfare issues, but also their significant economic consequences [51]. In general, skeletal deformities are a set of different complex bone disorders including vertebral and spinal malformations such as lordosis, scoliosis, and kyphosis, or platyspondyly (compressed vertebrae). Fish with a deformed mouth, fins, or vertebral axis are often characterized by impaired feeding and swimming behavior, consequently lowering feeding efficiency, growth rates, and resistance to stress and diseases. Many abiotic [52–54] and biotic [55, 56] factors affect the incidence of skeletal deformities, and certain nutrients in particular have been associated with their prevalence in different species [57–61]. The present study found that the Hydro feed induced only 6% skeletal deformities and reduced their overall occurrence by 17% compared to the Control diet. In Atlantic cod larvae, live food enriched with pollock (*Pollachius virens*) protein hydrolysate supported larval development and reduced occurrence of skeletal deformities compared to the control [62]. Peruzzi et al. [63] reported that dietary inclusion of fishmeal hydrolysate significantly reduced vertebral anomalies in triploid Atlantic salmon in comparison with the triploids fed solely an intact fishmeal-based diet which, as the authors speculated, was likely resulting from differences in phosphorus bioavailability between the two feed formulations. The reduction in skeletal deformities by dietary inclusion of protein hydrolysates seen in the present LMB study might also be associated with more efficient intestinal epithelial cell digestion and uptake of low molecular weight protein fraction (FAA and small peptides) as opposed to high molecular weight intact protein, leading to better nutrient availability for proper skeletal development [64]. To confirm this, postprandial FAA levels in LMB muscle were analyzed as indicators of dietary AA availability. The study found no significant differences in the levels of free IDAA, DAA, or total FAA in LMB muscle 3 hours after feeding between the two groups tested. However, the level of DAA, glycine, 3 hours after feeding was significantly lower in the Control versus Hydro group, while the concentration of IDAAs isoleucine, lysine, methionine, and valine showed an opposite trend, with levels significantly lower in the Hydro group compared to the Control group. It is generally recognized that all IDAA must be present simultaneously at appropriate levels in tissues to support optimal protein synthesis [65]. Since the Hydro group fish presented an improved growth performance (and hence increased muscle protein accretion), this result seemed counterintuitive. What is interesting to note is that the level of many of the FAAs, including arginine, glycine, methionine and valine, seemed to be at or below the basal FAA level found in 24-hour starved fish. The influx peak of FAA in the blood plasma and muscle varies depending on water temperature [66, 67] and fish digestive tract morphology (gastric versus agastric) [6, 9, 68].Since LMB is a stomach-possessing warm-water species, it is possible that assessment of FAA levels in the muscle 3 hours after feeding occurred at the point when the majority of the AA from the muscle FAA pool had already been utilized towards muscle protein synthesis, especially since absorption of AA from dietary hydrolysates is faster compared to non-hydrolyzed protein. In channel catfish (*Ictalurus punctatus*), blood plasma concentrations of alanine, arginine, aspartic acid, glutamic acid, glycine, histidine, leucine, lysine, serine, threonine, and valine reached the highest levels 1–3 h after a meal in fish fed diets where the protein source was composed of AA mixtures compared to fish that received feeds based on practical ingredients [69]. The appearance of AA transporters coincides with larval development in fish [70], and therefore a supply of dietary AA in larval fish can be ensured by peptides, which have their independent intestinal transporters [71]. Wei et al. [72] reported that high levels of dietary fish hydrolysate down‐regulated gene expression of the intestinal di- and tripeptide transporter PepT1. Our *in vitro* hydrolysis method yielded mostly low molecular weight compounds, and therefore it would also be reasonable to assume that that an excess of small size peptides led to “delayed” peptide absorption and muscle FAA peak in the bass fed the Hydro feed compared to the control. In general, very little is known about postprandial variations in AA concentration in tissues and how different proportions of individual IDAA and DAA may influence protein accumulation rates [73]. Amino acids are also a major source of energy, with 40% being catabolized in adults [74, 75] and a higher percentage in larval stages [33]. However, which AA are preferentially catabolized and how the proportions of all AA affect those catabolic rates is also not well understood.

The present study utilized digestives tracts from adult LMB to hydrolyze Asian carp muscle and obtain an optimal profile of muscle protein hydrolysates for inclusion in diets for larval LMB. For the first time we present methodology for obtaining a high-quality and well-utilized, pre-digested dietary protein source from Bighead carp which appears to be a practical way of producing, in a controlled way, an innovative and natural dietary ingredient for larval LMB diets and an effective way of utilizing Bighead carp as a starter feed ingredient source. Our hydrolysis method and later proteomic analysis proved that incubation of the substrate (muscle) with the supernatant obtained from fish digestive tracts allows for the breakage of complex intact muscle protein into lower molecular size products, creating a new opportunity for utilization of fish byproducts in the production of high-quality protein ingredients for larval fish feeds. This novel dietary ingredient appears to be characterized by the optimal molecular size range of the protein fraction that leads to positive growth responses in LMB larvae. Although the detailed molecular profile assessment of the hydrolysate was not part of the study, the present results encourage further investigation of the presented hydrolysis method and how time and/or enzyme/substrate levels can affect the molecular profiles of obtained hydrolysates. Furthermore, it seems imperative to establish the proteolytic activity dynamic in larval LMB digestive tract to advance development of a set of formulations varying in molecular fractions of peptides to match even further the status of the larval digestive tract development in terms of its digestive and absorptive capacity.

Overall, the results derived from the present study suggest that dietary protein hydrolysate for larval LMB obtained using our innovative *in vitro* hydrolysis method improves larval LMB growth performance. This unique diet formulation using Bighead carp muscle hydrolysate has the potential to lead to production of more robust and healthy larval fish with better adaptation capacity to formulated diets and therefore, a much easier weaning process (transition from live food to dry food). Consequently, these results should be used as solid preliminary data towards further optimization of fish muscle hydrolysates that will help reduce or completely eliminate the use of live food in larval LMB culture. We also believe that the method of using endogenous digestive enzymes to hydrolyze fish muscle could potentially be applied to other species characterized by larval size and developmental status similar to larval LMB.

## Supporting information

S1 File Growth and development analysis. This file contains the data for growth performance parameters and skeletal deformities.
(XLSX)

S2 File FAA composition. This file contains the data for the muscle FAA composition at 3 and 24 hrs post-feeding.
(XLSX)

S1 Raw images Original gel image. This file contains the original, uncropped image of the SDS-PAGE results.
(PDF)
