# Supplementary material for: Fish muscle hydrolysate obtained using largemouth bass Micropterus salmoides digestive enzymes improves largemouth bass performance in its larval stages
Source: PLoS One. 2021 Dec 28;16(12):e0261847. doi: 10.1371/journal.pone.0261847 (PMC8714084; doi:10.1371/journal.pone.0261847)
Supplement: S1 Raw images — This file contains the original, uncropped image of the SDS-PAGE results. (PDF) [file pone.0261847.s003.pdf]

Fish muscle hydrolysate obtained using Largemouth Bass *Micropterus salmoides* digestive enzymes improves Largemouth Bass performance in its larval stages

Karolina Kwasek[1*!], Christian Gonzalez[1], Macdonald Wick[2], Giovanni S. Molinari[1], and Michal Wojno[1!]

[1]Center for Fisheries, Aquaculture, and Aquatic Sciences, School of Biological Sciences, Southern Illinois University, Carbondale, IL
[2]Department of Animal Science, The Ohio State University, Columbus, OH

*Corresponding author; Email: karolina.kwasek@siu.edu; [!]Authors have equal contribution

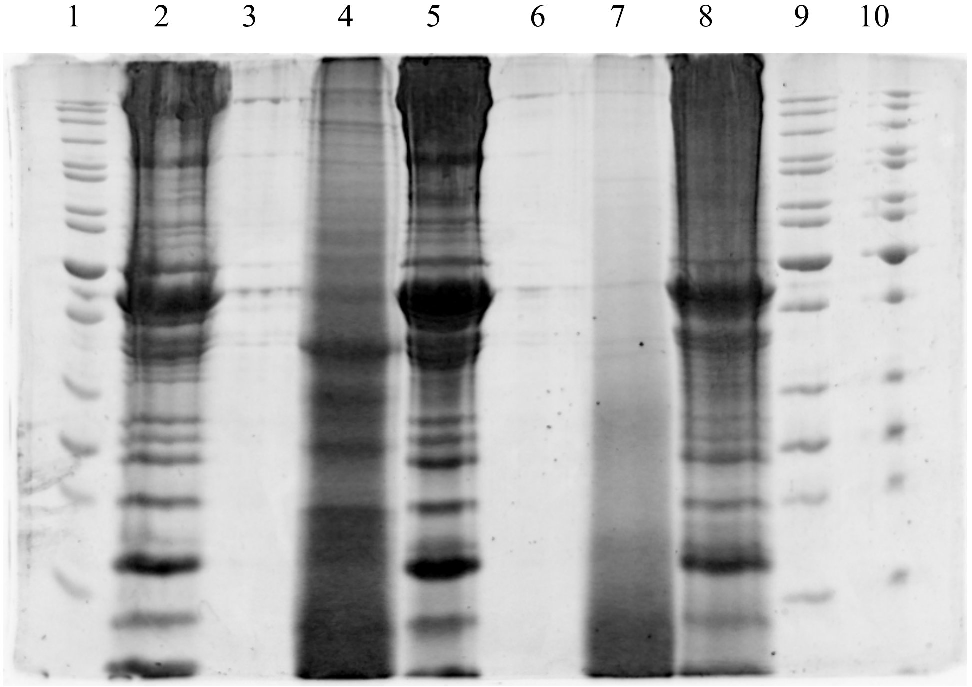

Figure 2. An uncropped image of 10% T denaturing SDS PAGE of Bighead carp muscle hydrolysate obtained using adult largemouth bass endogenous digestive enzymes. Each lane loaded with ~200 ug of protein. 1 – ladder protein ladder (marker; 200 – 10 kDa from top to bottom); 2 – control, muscle homogenate and enzymatic cocktail mix after heat shock, no enzymatic digestion; 3 – blank sample; 4 – stomach digestion, muscles enzymatically hydrolyzed in acid pH; 5 – blank intestinal digestion, muscles incubated in acid and alkaline pH without LMB digestive enzyme extract; 6 – blank sample; 7 – intestinal digestion, muscles enzymatically hydrolyzed in acid and alkaline pH; 8 – muscle incubated in acid pH without LMB digestive enzyme extract; 9-10 ladder protein marker.
